# Financial incentives for smoking cessation in pregnancy: a single-arm intervention study assessing cessation and gaming

**DOI:** 10.1111/add.12817

**Published:** 2015-02-26

**Authors:** Diana Ierfino, Eleni Mantzari, Julie Hirst, Tina Jones, Paul Aveyard, Theresa M Marteau

**Affiliations:** 1 Centre for the Study of Incentives in Health, King's College LondonLondon, UK; 2 Behaviour and Health Research Unit, University of Cambridge, Institute of Public HealthCambridge, UK; 3 Public Health Department, Derbyshire County CouncilDerbyshire, UK; 4 Derbyshire County Stop Smoking Service, Walton HospitalChesterfield, UK; 5 University of Oxford, Nuffield Department of Primary Care Health Sciences, Radcliffe Observatory QuarterOxford, UK

**Keywords:** Financial incentives, gaming, pregnancy, smoking, smoking cessation, vouchers

## Abstract

**Aims:**

Financial incentives were the single most effective intervention for smoking cessation in pregnancy in a recent Cochrane Review, but based on a few small trials in the United States using only 7-day point prevalence measures of cessation. This study estimates (a) prolonged cessation in an unselected population of English pregnant smokers who are offered financial incentives for quitting and (b) ‘gaming’, i.e. false reporting of smoking status to enter the scheme or gain an incentive.

**Design:**

Single-arm intervention study

**Setting:**

Antenatal clinic and community

**Participants:**

A total of 239 pregnant smokers enrolled into the financial incentive scheme, attending for maternity care at one hospital in an area of high deprivation in England over a 42-week period.

**Measurements:**

Smoking cessation at delivery and 6 months postpartum, assessed using salivary cotinine; gaming assessed using urinary and salivary cotinine at enrolment, 28 and 36 weeks gestation, and 2 days and 6 months postpartum.

**Findings:**

Thirty-nine per cent (239 of 615) of smokers were enrolled into the scheme, 60% (143 of 239) of whom made a quit attempt. Of those enrolled, 20% [48 of 239; 95% confidence interval (CI) = 14.9%, 25.1%] were quit at delivery and 10% (25 of 239; 95% CI = 6.2%, 13.8%) at 6 months postpartum. There was no evidence that women gamed to enter the scheme, but evidence that 4% (10 of 239) of those enrolled gamed on one or more occasions to gain vouchers.

**Conclusions:**

Enrolment on an incentive scheme in an unselected English cohort of pregnant smokers was associated with prolonged cessation rates comparable to those reported in US trials. Rates of gaming were arguably insufficiently high to invalidate the use of such schemes.

## Introduction

Smoking in pregnancy is a major cause of adverse infant outcomes, including death [1–3]. While the prevalence of smoking in pregnancy has declined, it remains high in those who are more socially deprived. For example, in England, 12% of women smoke throughout pregnancy, ranging from 0.5% in areas of low deprivation to 27% in areas of high deprivation [4].

A recent Cochrane Review found financial incentive schemes to be the single most effective intervention for smoking cessation in pregnancy, with an estimated quit rate of 24% for those offered incentives compared with 6% for those receiving other interventions [5]. The promising use of financial incentives for promoting smoking cessation and improving birth outcomes has also been confirmed by a recent analytical review focusing specifically on this intervention [6], as well as by a meta-analysis of the three most robust trials in the Cochrane review [7]. These trials, however [8–10], included only 350 women in total and used only 7-day point prevalence of smoking abstinence as a primary outcome, leading authors to recommend replication in more robust designs, using larger samples and standardized assessments of continuous abstinence. Also, the findings from these trials are not informative about the use of financial incentives in routine practice.

World-wide, there are few reports examining the use financial incentive schemes in routine practice for smoking cessation in pregnancy. One programme conducted in Scotland reported 20% of identified smokers engaging with the scheme, of whom 32% were quit at 12 weeks after joining the scheme and 17% at 3 months postpartum. Considering all smokers in the area where the scheme was running, 4% were quit at delivery [11]. Smoking status, however, was ascertained using carbon monoxide (CO) breath tests, which only assesses smoking in the preceding few hours, and could thus overestimate quit rates. This is a particular issue in studies of incentives, which could lead to ‘gaming’ (i.e. false reporting of smoking status). There are two types of ‘gaming’: first, to gain entry into the scheme (i.e. non-smokers acting as smokers for example, by smoking a cigarette prior to a CO test), and secondly, to gain an incentive (i.e. smokers acting as non-smokers). We are unaware of any studies that have considered the first form of gaming and only one study which provides some evidence for the second form: when the smoking status of incentivized participants was verified using CO, 37% were classified as abstinent, compared with 7% when cotinine was used (equivalent figures in the control group were 8% and 5%) [12], suggesting that four in five people who claimed abstinence were not really abstinent. A further adverse effect of offering financial incentives to pregnant smokers is that they may lead women to delay initiating a quit attempt until enrolment on the scheme. We are unaware of any study that has examined this.

We report here a single-arm intervention study aiming to assess the potential effectiveness of financial incentives for smoking cessation in pregnancy and to inform the use of incentive schemes in routine clinical practice. The study was designed to address two key uncertainties concerning the use of financial incentives for smoking cessation during pregnancy: its effectiveness in unselected populations and the extent to which it leads to ‘gaming’. The latter has been a particular concern for service planners, policy makers and the public, standing as a significant barrier to the implementation of incentive schemes, which have the potential to increase quitting in pregnant smokers [13]. In addition, the study aimed to examine the role of certain predictors of participation in the scheme and smoking cessation. Of particular interest is the extent to which financial incentive schemes achieve greater quitting for those most socially deprived. A recent meta-analysis of the impact of financial incentives on several health-related behaviours, including smoking, found behaviour change with incentives to be greater in more deprived populations [14]. This contrasts with the observational study in Scotland, described above, in which quit rates at 4 weeks and delivery were higher for less deprived women [11]. Of further interest is the extent to which financial incentives achieve greater quitting in those who discount the future more steeply, i.e. prefer smaller, more immediate rewards over larger, later ones. One of the possible psychological mechanisms by which incentives may change behaviour is by providing an immediate reward for engaging in a behaviour with normally distant benefits. Pregnant smokers have several characteristics associated with steeper discounting of the future in addition to being smokers, namely being younger, having less education and lower income compared with pregnant non-smokers [15,16].

### Study aim and objectives

The specific study objectives are:

1. To estimate the proportion of pregnant women who smoke at the point of receiving antenatal care at one hospital over a 42-week period and who accept the offer to participate in a financial incentive scheme for smoking cessation.
2. To estimate the proportion of pregnant smokers who initiate a quit attempt on the scheme and who achieve prolonged abstinence at (a) delivery and (b) 6 months postpartum.
3. To estimate the prevalence of two sets of adverse outcomes of using financial incentives schemes for smoking cessation:
  - delay in quitting, in order to enrol in the scheme;
  - gaming, i.e. false reporting of smoking status:
  - to gain entry to the scheme
  - to gain an incentive
4. To examine predictors of (a) participation in the scheme and (b) smoking cessation at delivery and 6 months postpartum

## Methods

Full details of the study methods are presented elsewhere [17]. The study was approved by the Derbyshire Research Ethics Committee (Ref. no.11/H0401/2), which included permission to use limited data on patients who did not give consent, in accord with the Data Protection Act (1998).

### Design

Single-arm intervention study.

### Participants

Participants included all pregnant smokers attending a first antenatal clinic appointment at one hospital in Chesterfield England between 14 November 2011 and 31 August 2012. Those eligible for inclusion were pregnant women who reported smoking and/or had CO readings of >6 parts per million (p.p.m.) and a urinary cotinine concentration of ≥1.5 ng/ml. Excluded were women who: were unable to provide informed consent; were aged less than 16 years of age; or did not speak English. We recruited sufficient women to provide an estimate of quitting in the range of that observed in published trials (24%) with a precision of ± 7% (for further details, see [17]).

### The intervention

The scheme involved provision of a shopping voucher upon CO validation of self-reported abstinence. The size of incentives increased by £1 for each visit at which smoking cessation was confirmed, from the first voucher (worth £8) to the last voucher (worth £39), providing a maximum total of £752-worth of vouchers. The size of the incentive is similar to that offered in the three randomized controlled trials included in the aforementioned Cochrane Review, each of which reported large effects on abstinence [8–10]. Testing positive for smoking resulted in the incentive being withheld at that visit and the value being reset to baseline (£8) for the next visit. Following two consecutive test results indicating no smoking, the incentive value was reset to the highest point attained prior to the lapse. Up to 32 visits were possible from early pregnancy until 6 months postpartum (up to 16 during pregnancy and 16 postdelivery), occurring most frequently in the first 2 weeks of participation and the first 2 weeks after birth and then decreasing in frequency until delivery and 6 months postpartum, respectively (see Table2 of the protocol [17] for details of the incentive schedule).

### Measures

#### Smoking cessation

Smoking abstinence was classified in line with the Russell standard criteria [18]. At delivery it was operationalized by CO-confirmed self-reported complete smoking abstinence between 6 weeks after enrolment to 36 weeks gestation, validated by salivary cotinine of <15 ng/ml at 36 weeks. For women using nicotine replacement therapy, anabasine, a tobacco-specific alkaloid, was analysed instead of cotinine. All women lost to follow-up were assumed to have resumed smoking. Smoking cessation 6 months postpartum was operationalized in the same way for the time-period between 2 days and 6 months postpartum.

#### False reporting of smoking status

- To enter the scheme as a non-smoker while pretending to be a smoker
  This was operationalized by self-report of smoking, validated by CO levels compatible with smoking, but with urinary cotinine measurement at enrolment compatible with not smoking (<1.5 ng/ml).
- To receive incentives on the scheme while continuing to smoke

This was operationalized by self-report of not smoking, validated by CO levels compatible with not smoking, but salivary cotinine, collected at 28 and 36 weeks gestation, and 2 days and 6 months postpartum, compatible with smoking (>15 ng/ml). For women using nicotine replacement therapy, anabasine was analysed. Women were not warned in advance about these tests, although they were aware that they needed to pass the CO test to claim the voucher. Women proved to be smoking on any of these extra tests (i.e. those using saliva as opposed to CO) were not confronted with this, withdrawn from the scheme or refused vouchers. The results of these tests were known only to the research team and none of those administering the scheme.

#### Predictors of smoking cessation

Delay discounting was assessed using a single-item measure requesting participants to choose between £45 in 3 days or £70 in 3 months [15]. Nicotine dependence was measured using the Heaviness of Smoking Index (HSI) [19], which assesses the amount of cigarettes smoked and the time of the first cigarette after waking. Socio-economic status was assessed using education as an individual-level measure, and postcodes to generate an area-level measure (Index of Multiple Deprivation (2010) [20]).Women's ages were also recorded.

### Procedure

Midwives at the study hospital sent details of any woman at booking reporting smoking and/or having a CO reading of >6 p.p.m. to the Derbyshire Community Health Service (DCHS) Stop Smoking Service (SSS), in keeping with existing standard care. Midwives gave smokers two leaflets: one outlining the incentive scheme and one the SSS. The SSS provides behavioural support, including the use of cognitive–behaviour therapy and motivational interviewing to help smokers quit. Pregnant smokers are offered a 12-week course, which includes the option of being prescribed nicotine replacement therapy for the full 12 weeks. All pregnant smokers have a weekly one-to-one session of up to an hour with a specialist pregnancy adviser.

A support worker from the SSS telephoned all smokers within 2 working days of receipt of their details. Pregnant women who met the inclusion criteria for the incentive scheme were invited to enrol following a home visit. Those agreeing to this were posted an information sheet to read prior to the enrolment visit.

At the enrolment visit, the support worker provided women with an information sheet and answered potential questions about the scheme. Women completed a questionnaire to assess predictors of smoking cessation and gave signed consent for participation in the scheme and collection and analysis of samples for cotinine estimation (two at 28 and 36 weeks gestation, and two at 2 days and 6 months postpartum).

A urine sample was taken and tested at this visit to assess eligibility for the scheme, with urinary cotinine concentration ≥1.5 ng/ml validating smoking and hence eligibility for the scheme.

All women enrolling on the incentive scheme were offered the support of the NHS SSS.

### Data analyses

Descriptive statistics were used to present the main outcomes regarding proportions of women who enrolled into the scheme, initiated a quit attempt and were quit at delivery and 6 months postpartum. Logistic regression was used to predict the latter three outcomes. The predictors for the first outcome were: socio-economic status (area-level) and age, the only two variables available for those not enrolling. The predictors for the three other outcomes were: age, socio-economic status (assessed at individual level and area level), nicotine dependency and delay discounting. A parallel set of regressions was conducted for the two quit outcomes with different comparison groups: (i) with those enrolled but not quitting; and (ii) with those achieving the preceding quitting step (initiating a quit attempt or quitting at delivery), but failing to make the next step (quitting at delivery or 6 months postpartum).

## Results

**Figure 1:**
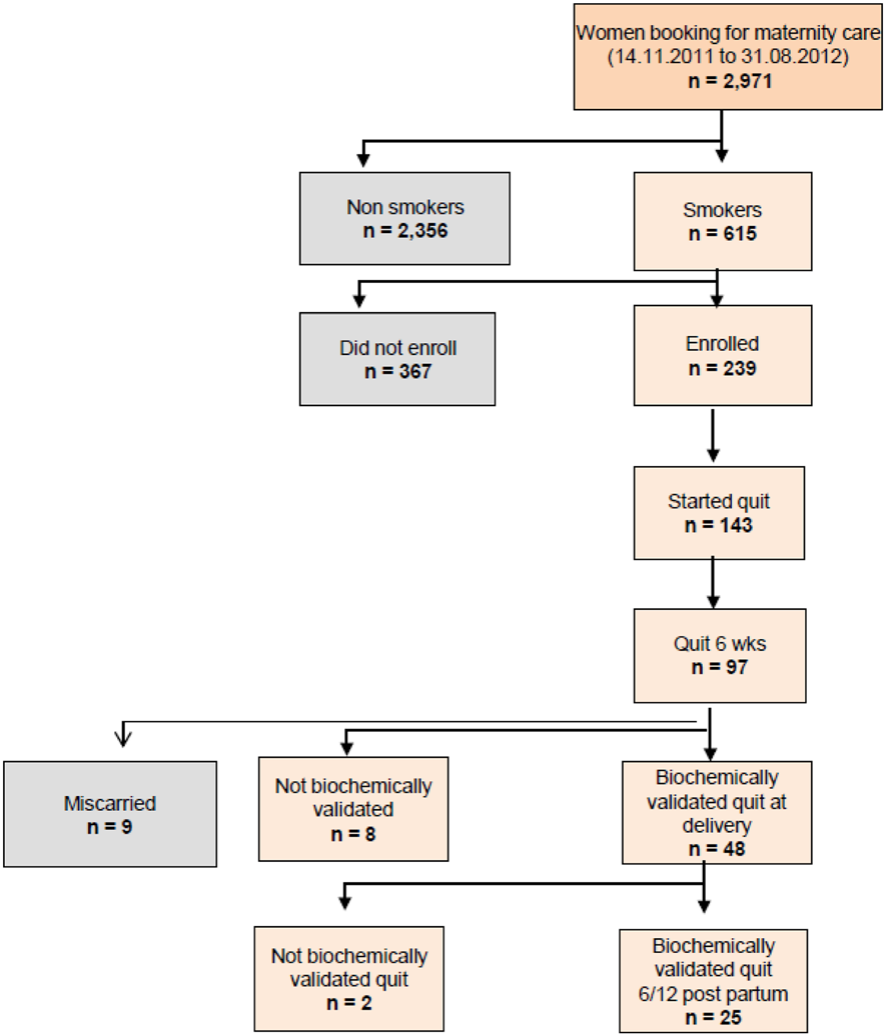
Flowchart showing smoking-related outcomes for the cohort of women offered participation in the scheme

During the study period, 2971 women attended antenatal care at the study hospital, of whom 615 reported smoking (21%). The smoking-related outcomes for this cohort are shown in Fig. 1.

### 

#### Enrolment in the financial incentive scheme

A total of 239 of the 615 smokers enrolled into the scheme (39%). Those who enrolled were of similar age and deprivation level to those who did not enrol (Table1).

**Table 1:** Baseline characteristics of women who did and did not enrol into the scheme.^a^

	*Enrolled (n = 239)*	*Not enrolled (n = 367)*
Age (mean ± SD)	25.2 (5.7)	25.7 (5.9)
Nicotine dependence (mean ± SD)	1.8 (0.9)	NA
*Socio-economic status (SES)*		
Area level (IMD: % low SES)	178/239 (75%)	248/367 (68%)
Individual level (education: % low SES)	86/239 (36%)	NA
Delay discounting	123/239	NA
(% less now versus more later)	(51%)	

a Those who enrolled were of similar age to those who did not enrol (25.2 and 25.7 years, respectively: t(597) = 1.08, *P* = 0.85). Enrolees and non-enrolees were also similar in deprivation, with 74% of the former and 73% of the latter being in the most deprived tertile [χ ^2^ (d.f. = 1, *n* = 579) = 0.35, *P* = 0.85]. Logistic regression analyses confirmed that age and deprivation level did not predict enrolment into the scheme. NA = not available; SD = standard deviation; IMD = index of multiple deprivation.

#### Initiating a quit attempt

A total of 143 of those who enrolled into the scheme (60%) made a quit attempt, i.e. received at least one voucher for smoking cessation. The chances of making a quit attempt on the scheme decreased in women who were most deprived compared to those who were less deprived (Table2).

**Table 2:** Predictors of smoking related outcomes.

	*Initiated attempt*				*Quit at delivery*				*Quit at 6 months postpartum*			
			
			*Univariate analysis*	*Multivariable analysis*			*Univariate analysis*	*Multivariable analysis*			*Univariate analysis*	*Multivariable analysis*
												
	*Yes n = 143*	*No n = 96*	*OR (95% CI)*	*OR (95% CI)*	*Yes n = 48*	*No n = 191*	*OR (95% CI)*	*OR (95% CI)*	*Yes n = 25*	*No n = 214*	*OR (95% CI)*	*OR (95% CI)*
*Age* (mean ± SD)	25.9 (5.8)	24.3 (5.6)	1.05 (1.00–1.10)	1.05 (0.99–1.10)	26.4 (5.8)	24.9 (5.7)	1.04 (0.98–1.10)	1.02 (0.96–1.09)	28.0 (5.6)	24.9 (5.7)	1.09 (1.02–1.17)	1.07 (0.99–1.16)
*Nicotine dependence* (mean ± SD)	1.8 (0.9)	1.8 (0.9)	1.01 (0.75–1.35)	0.97 (0.71–1.33)	1.8 (1.04)	1.8 (0.9)	1.05 (0.74–1.50)	1.03 (0.71–1.49)	1.9 (1.04)	1.8 (0.9)	1.02 (0.63–1.63)	0.94 (0.56–1.58)
*Socio–economic status (SES)*												
Area level (IMD): % low SES	100/143 (70%)	78/96 (81%)	0.54 (0.29–1.00)	0.46* (0.22–0.96)	32/48 (67%)	146/191 (76%)	0.62 (0.31–1.22)	0.71 (0.34–1.51)	14/25 (56%)	164/214 (77%)	0.39* (0.17–0.91)	0.47 (0.18–1.23)
Area level (IMD): % high SES	43/143 (30%)	18/96 (19%)			16/48 (33%)	45/191 (76%)			11/25 (44%)	50/214 (23%)		
Individual level (education): % low SES	46/132 (35%)	40/82 (49%)	0.55 (0.31–0.96)	0.68 (0.38–1.23)	11/44 (25%)	75/169 5(44%)	0.42* (0.19–0.88)	0.45* (0.21–0.97)	3/22 (14%)	83/191 (44%)	0.20* (0.59–0.72)	0.26* (0.73–0.94)
Individual level (education): % high SES	86/132 (65%)	41/42 (51%)			33/44 (75%)	94/169 (56%)			19/22 (86%)	108/191 (56%)		
*Delay discounting*												
% Less now versus more later	73/136 (54%)	50/85 (59%)	0.81 (0.47–1.40)	0.88 (0.49–0.58)	23/46 (50%)	100/175 (57%)	0.75 (0.39–1.44)	0.86 (0.43–1.69)	10/22 (46%)	113/199 (57%)	0.63 (0.26–1.54)	0.73 (0.28–1.87)
% More later versus less now	63/136 (46%)	35/85 (41%)			23/46 (50%)	75/175 (43%)			12/22 (54%)	86/199 (43%)		

* Significant at the 5% statistical significance level. IMD = index of multiple deprivation; SD = standard deviation; CI = confidence interval; OR = odds ratio.

#### Smoking cessation at delivery

Forty-eight women were confirmed as sustained quitters at delivery: 20% (48 of 239, 95% confidence interval (CI)=14.9%, 25.1%) of those enrolled into the scheme, 34% (48 of 143) of those who initiated a quit attempt and 8% (48 of 615) of the total cohort of smokers.

Comparing those quit at delivery with those enrolled who did not quit, the chances of being quit at delivery decreased in women who were more deprived compared to those who were less deprived (Table2).

Comparing those quit at delivery with those who initiated a quit attempt but did not quit, the chances of being quit at delivery were not predicted by any of the independent variables.

#### Smoking cessation at 6 months postpartum

Twenty-five women were confirmed as sustained quitters at 6 months postpartum (23 of whom were quit at delivery^1^), 10% (25 of 239, 95% CI=6.2%, 13.8%) of those enrolled into the scheme, 17% (25 of 143) of those who initiated a quit attempt and 4% (25 of 615) of the total cohort of smokers.

Comparing those quit at 6 months postpartum with those who enrolled but were not quit at this time, the chances of being quit at 6 months postpartum decreased in women who were more deprived compared with those who were less deprived (Table2).

Comparing those quit at 6 months postpartum with those who were quit at delivery but not at 6 months postpartum, the chances of smoking cessation were not predicted by any of the independent variables.

#### Adverse outcomes of using financial incentives schemes for smoking cessation

- Delay in quitting, in order to enrol in an incentive scheme
  This was assessed indirectly by comparing the proportion of pregnant smokers identified during the study with the proportion of smokers attending the same hospital in similar time-periods in the preceding 2 years. During the study period, 21% (615 of 2971) of women at their first hospital visits were recorded as smokers compared with 18% in the equivalent time-period in 2010/2011 (423 of 2340) [χ^2^ = 3.86; *P* = 0.049]; and 18% in 2009/2010 (386 of 2125) and [χ^2^ = 3.40; *P* = 0.065].
- Gaming, i.e. false reporting of smoking status
  - To gain entry to the scheme (i.e. non-smokers acting as smokers)
    All tests of urinary cotinine taken during enrolment were positive for smoking, suggesting that no women gamed to enter the scheme.
  - To gain an incentive (i.e. smokers acting as non-smokers)

Ten of the 239 [4%; 95% confidence interval (CI) = 1.5%, 6.5%] women enrolled into the scheme, comprising 7% of those making a quit attempt, had at least one salivary cotinine or anabasine result compatible with smoking having reported not smoking and simultaneously generating a CO reading compatible with not smoking.

## Discussion

Of the 39% of smokers who enrolled into the scheme, one in five had quit smoking at delivery, and one in 10 at 6 months postpartum. When considering the complete cohort of smokers identified during the study period, 8% were quit at delivery and 4% at 6 months postpartum. Those who succeeded in quitting were generally less deprived than those who did not. There was evidence that 4% of the women who enrolled ‘gamed’ the system by acting falsely as non-smokers.

The quit rates achieved on the incentive scheme were higher than those achieved in a comparable period in the preceding year at the same hospital (0%), and similar to those reported in a recent Cochrane Review of smoking cessation in pregnancy, which estimated a quit rate at delivery with incentives of 24% of all women enrolled into an incentive scheme [5]. A recent pilot study in Scotland reported that 17% of women enrolled into an incentive scheme were abstinent 3 months after delivery [11], although abstinence was not validated using the Russell criteria, so quit rates may have been overestimated.

The proportion of eligible participants who enrol in smoking cessation programmes is an important index of potential population impact. In the current study, two in five identified smokers enrolled into the scheme, a proportion which could have potentially increased with more assiduous attempts to contact all smokers.^2^ None the less, enrolment rates were higher than those achieved in the aforementioned Scottish study, which reported one in five smokers enrolling [11]. The proportion of those enrolled who made a quit attempt was 60%. While those enrolling did not differ from those not enrolling in terms of social deprivation, those enrolled who made a quit attempt were less deprived. Providing additional support to those who enrol but do not initiate a quit attempt may prove effective in increasing those initiating a quit attempt, as well as reducing the social gradient in those initiating and sustaining a quit attempt.

The study hospital served an area of England with high levels of deprivation, as well as higher than average rates of smoking in adults and pregnant women [21]. Accordingly, three in four of women enrolled into the scheme were in the most deprived tertile. Those enrolling were no less deprived than those not enrolling, in keeping with previous studies reporting that motivation to stop smoking is unrelated to socio-economic status [22,23]. Success at quitting is, however, related to deprivation, as observed in the current study and others [24,25]. There are several possible explanations for the robust finding that those who are most deprived are least likely to quit. The first is that this reflects the higher levels of delay discounting observed in those who are more deprived [15]. Delay discounting, however, was unrelated to cessation in the current study, suggesting that the adverse impact of deprivation on quitting was not due to high delay discounting. It is possible that the offer of financial incentives might have ameliorated the normally detrimental impact of women's preference for the present, by providing immediate rewards for a behaviour with typically delayed benefits. An alternative explanation is that those who are more deprived have higher levels of nicotine dependence [26], which can explain some of the variation in the social pattern of quitting [27]. In the current study, however, nicotine dependence was unrelated to quitting success. Another explanation is that in deprived individuals the brain systems that control behaviour are weaker, leading to a reduced ability to sustain goal-directed behaviour, such as quitting smoking, and in particular an inability to inhibit responses that may be cued by cravings or exposure to others smoking [28]. The latter possibility highlights the role of various social and physical environmental factors in the reduced successes of those who are deprived that were not assessed in the current study. These include a lack of social support in quitting among those who are more deprived [24], increased exposure to smoking (with attendant influence on social norms and mirror neurones), given the higher prevalence of smoking among socially deprived groups, and the possible increased density of tobacco retailers in areas of higher deprivation, which can reduce the success of quitting [29].

We investigated three forms of gaming: smokers delaying quitting until enrolling into the scheme; non-smokers acting as smokers to gain entry into the scheme; and smokers acting as non-smokers to gain rewards for quitting. We found weak evidence for the first form of gaming, in that there were more pregnant smokers attending for antenatal care during the study period than in the equivalent period during the 2 preceding years. As we have noted previously, using historical controls to infer change is subject to a number of biases [17]. The most likely explanation for this difference in prevalence of smoking in the two periods is that the study period coincided with the introduction of routine CO testing for all pregnant women, in keeping with National Institute for Health and Care Excellence (NICE) guidelines on caring for pregnant smokers [30]. Reliance on self-report of smoking during pregnancy underestimates rates of smoking [31]. There was no evidence that women gamed to enter the scheme. This would have required women to know about the scheme in advance of attending a first antenatal appointment, and knowing that smoking just one cigarette in the hours prior to the appointment was sufficient for a reading on the CO monitor compatible with smoking. The incentive scheme had received wide publicity through a local newspaper, as well as radio and television. Information about the scheme might have also spread by word of mouth. Methods for ensuring a positive or a negative test result for smoking on CO monitors are available on the internet, so it was plausible that some non-smokers might have gamed the system, but none did, according to our assessments. There was, however, evidence that 7% of those making a quit attempt (comprising 4% of those enrolled into the scheme) received incentives on one or more occasions when they were continuing to smoke. While women were not reminded in advance of scheduled visits when saliva samples would be taken for cotinine validation, it is possible that some may have recalled from their enrolment visits (at 12 weeks) when in pregnancy these would be taken (28 and 36 weeks gestation and 2 days and 6 months postpartum). While we think this is unlikely, an even more robust estimate of gaming during pregnancy would be provided from cotinine analyses of routinely collected urine or blood samples. These are not generally available postpartum. Had the study relied upon self-report and CO tests alone, the proportion quit on the scheme would increase from 20% to 23% and from 8% to 9% of the total population. The rates of incongruence between self-reported smoking status and cotinine results are comparable to those reported in prior efficacy trials [32]. These effects are arguably modest in their impact on estimates of effectiveness of financial incentives for smoking cessation. We estimated that between £2649 (if we assume some women were quit during some parts of the scheme) and £3607 (if we assume women who gamed did so throughout the entire scheme) was paid out to women who continued to smoke, between 7% and 9.6% of £37 490, the total amount spent on incentives.

### 

#### Strengths and limitations of the current study

This study is, to our knowledge, the first to assess the nature and scale of gaming in a robust and systematic way. It therefore makes a useful contribution to service planners and policy makers (as well as the public) who are keen to consider financial incentive schemes for pregnant smokers but have been reluctant, assuming that the scale of deception is far higher than that observed in the current study. This is also one of the few studies to assess the impact of introducing an incentive scheme into routine practice as part of antenatal care, thereby providing an estimate of its population impact in an unselected cohort of pregnant smokers.

The study had several limitations. First, it was a single-arm intervention study using historical controls against which to estimate incentive effectiveness. An experimental design would provide much stronger evidence of effectiveness of the scheme. Given our study aims and the resources available to us, we opted to run this study as a single-arm intervention to inform the interpretation of existing randomized controlled trials and the design of future trials. Second, it was run as part of routine care, and lacked the resources to ensure that three attempts were made to contact all identified smokers. Third, data on miscarriage rates were not collected, the absence of which will have resulted in a small underestimate of quit rates in the observed cohort. Additionally, the incentive scheme involved up to 32 contacts for women. Other incentive schemes have been run with far fewer contacts, offering larger incentives at less frequent intervals [33]. Future research is needed that assesses the impact of incentive scheme characteristics on effectiveness, including magnitude, frequency and type of incentives.

#### Implications

This intervention was run with one additional employee who worked with the existing public health smoking cessation service. No formal cost-effectiveness analyses were conducted. The amount spent on incentives was £37 490. The cost-effectiveness of this and similar schemes remains to be assessed formally. The costs of the present scheme were estimated to be £139 500. Presenting the effectiveness in terms of 23 (48–25) women who did not smoke during the last 28 weeks of their pregnancies and the 25 women who stopped smoking until at least 6 months postpartum, this is likely to prove an acceptable cost : benefit ratio. Based on modelling of other interventions for smoking cessation in pregnancy, it is most likely that these schemes fall within the acceptable range of cost-effectiveness set by NICE [34].

We made no formal evaluation of how the scheme was received at institutional levels. We spent time discussing the scheme with managers and clinicians, some of whom expressed reluctance. Any initial reluctance was reduced markedly when information on potential effectiveness was presented, in keeping with the research evidence [35]. Future research might also address how best to increase recruitment of pregnant smokers to an incentive scheme, how to encourage those enrolled to initiate a quit attempt and how quit rates for deprived women might be increased.

## Conclusions

In a cohort of unselected pregnant women, two in five smokers enrolled in an incentive scheme for smoking cessation. Of these, one in five were quit by delivery and one in 10 by 6 months postpartum. These cessation rates compared favourably with those of historical controls attending the same hospital for antenatal care in the preceding year, in which 0% were recorded as having stopped smoking. Approximately one in 25 of the women enrolled into the scheme presented themselves falsely on one or more occasions as non-smokers. These rates of gaming are arguably insufficiently high to invalidate the use of such schemes.

## Footnotes

1 Two women were quit at 6 months but not at delivery. Both reported being quit at delivery, but their smoking status could not be verified with cotinine. For one woman, a saliva sample was collected but lost. For the other woman, insufficient saliva was collected to allow testing.

2 The limited resource available in the current study prevented us from achieving our target of making three attempts to contact each of the 615 smokers.
